# Silanization of Starch and Its Effect on Cross-Linking and Mechanical, Dynamic, Hydrophobic, and Aging Properties of Polymeric Compositions Containing Natural Rubber

**DOI:** 10.3390/ma17246273

**Published:** 2024-12-22

**Authors:** Konrad Mrozowski, Aleksandra Smejda-Krzewicka

**Affiliations:** Institute of Polymer and Dye Technology, Faculty of Chemistry, Lodz University of Technology, Stefanowskiego Street 16, 90-537 Lodz, Poland

**Keywords:** natural rubber, starch, silanization, hydrophobicity, contact angle, bio-composite

## Abstract

In recent years, the search for more sustainable fillers for elastomeric composites than silica and carbon black has been underway. In this work, silanized starch was used as an innovative filler for elastomeric composites. Corn starch was chemically modified by silanization (with n-octadecyltrimethoxysilane) via a condensation reaction to produce a hydrophobic starch. Starch/natural rubber composites were prepared by mixing the modified starch with elastomer. The morphology, hydrophobicity, and chemical structure of starch after and before modification were studied. The results showed that starch after silanization becomes hydrophobic (θ_w_ = 117.3°) with a smaller particle size. In addition, FT-IR spectrum analysis confirmed the attachment of silane groups to the starch. The modified starch dispersed better in the natural rubber matrix and obtained a more homogeneous morphology. The composite achieved the best dynamic (ΔG′ = 203.8 kPa) and mechanical properties (TS_b_ = 11.4 MPa) for compositions with 15 phr of modified starch. In addition, the incorporation of silanized starch improved the hydrophobicity of the composite (θ_w_ = 117.8°). The higher starch content allowed the composites to achieve a higher degree of cross-linking, resulting in better resistance to swelling in organic solvents. This improvement is due to enhanced elastomer–filler interactions and reduced spaces that prevent solvent penetration into the material’s depths. The improved mechanical properties and good dynamic properties, as well as improved hydrophobicity, were mainly due to improved interfacial interactions between rubber and starch. This study highlights the potential and new approach of silane-modified starch as a sustainable filler, demonstrating its ability to enhance the mechanical, dynamic, and hydrophobic properties of elastomeric composites while supporting greener material solutions for the rubber industry.

## 1. Introduction

Elastomeric composites based on natural rubber (NR) are used in areas such as automotives as a component of tires, gaskets, and dampers and in industry as conveyor belts, adhesives, or elastic bands. In addition, it is worth mentioning applications such as gloves, footwear coatings, and seals. Natural rubber has many desirable properties, including elasticity, resilience, and tensile strength, as well as water repellency. It also exhibits several disadvantages; thus, its use in specific applications is often limited [1,2,3]. One of the factors limiting the use of natural rubber as a coating material is thermo-oxidative or UV-induced aging. Both processes cause the splitting of the polymer chain, loss of elasticity, deterioration of mechanical properties, and increasing hydrophilicity [4]. In addition, during cross-linking of the elastomeric composite, significant reversion occurs, leading to a loss of elasticity and an increase in the brittleness of the material [5]. In solvent-affected environments, natural rubber tends to swell, which poses challenges for applications such as seals, coatings, or gaskets [3,6]. For this reason, different types of fillers are incorporated into the rubber to impart unique properties to the composite but also to compensate for its disadvantages related to aging or deterioration of mechanical and dynamic properties. Therefore, improving the performance of NR vulcanizates remains to this day a challenge for researchers to overcome many problems in specialized applications.

The two main large-scale fillers used in elastomeric composites are carbon black and silica [7,8]. However, these fillers are associated with high CO_2_ emissions and non-renewability. Because of the growing demand for sustainable materials and increasingly strict carbon footprint and carbon emission requirements, the search for natural alternatives to silica and carbon black is inevitable. Due to its high commonness, low price, and wide range of modification abilities, starch has captured the attention of researchers and industry [9,10]. It is a renewable and biodegradable polymer produced by many plants as a source of energy storage. It is extracted from natural sources such as cereals (corn, wheat, and some varieties of rice) and the roots and tubers of potatoes, cassava, or tapioca. Chemically, starch is a polysaccharide, containing two main fractions—amylose and amylopectin [11,12,13]. Amylopectin is the main component of starch, depending on its origin, and can account for 75% to 80% of its weight. This compound is a branched molecule consisting of thousands of glucose units that are linked together by α-(1→4) and α-(1→6) glycosidic bonds [14,15]. The other main building block of starch is amylose. Its content is 20–25% by weight, depending on its origin. Amylose is an unbranched fraction soluble in warm water, which consists of up to 5–600 glucose units linked together by α-(1→4) glycosidic bonds forming a helix structure. According to the arrangement of bonds and the content of individual components, starch has different physicochemical, thermal, and rheological properties [16,17].

Starch and elastomeric bio-composites containing natural rubber fit well as naturally derived materials and can be classified as sustainable. The use of starch instead of conventional fillers is an important advantage highlighted in these studies [18,19,20,21,22]. However, the use of starch as a filler also has many disadvantages, such as a large particle size, strong polar surface area, high cohesion energy, and high melting point [23,24]. These are among the barriers to using starch as a filler on a large scale in the rubber industry. Moreover, native starch tends to swell when exposed to polar solvents and readily absorb water, which can plasticize the rubber composite; reduce interfacial adhesion; and lead to swelling, loss of mechanical strength, and poor dimensional stability. In addition, starch particles tend to form clusters in the rubber matrix, leading to uneven distribution and suboptimal reinforcement [22,25]. A necessity for starch to be used successfully as a filler in NR composites is its physical or chemical modification.

**Figure 1:**
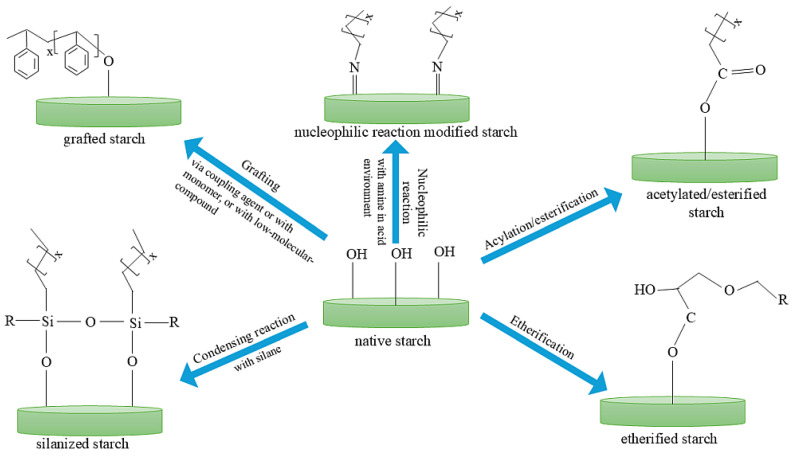
Common hydrophobic chemical modifications of starch.

Among the available literature, examples of starch treatments include physical modifications such as thermal treatment (gelatinization, cold water swelling, heat and moisture treatment, or annealing) [26,27,28]. In addition, it is worth mentioning non-thermal treatments, which include sonication, grinding, freeze-drying, or exposure to an electric field [29,30,31], and chemical modifications, such as acetylation, alkylation, silanization, acid hydrolysis, oxidation, and cross-linking [32,33,34,35,36]. Of the various chemical modifications affecting the structure and properties of starch, the grafting reaction has gained great popularity in recent years. It allows for the development of improved mechanical properties but also results in improved interactions at the filler–matrix interface [37,38,39,40]. The most popular modification method is the grafting of starch surfaces with low molecular weight compounds. By referring to the literature, silanization and reactions of esterification or acetylation of hydroxyl groups are worth mentioning [34,36,41,42,43]. All these modifications affect the morphology and properties of starch. Each of these methods has its advantages and disadvantages, so it is important to choose the optimal one for a given application. From the perspective of using starch as an elastomeric matrix filler, it is important to get rid of as many hydroxyl groups as possible to lower the surface energy for good dispersion. For this purpose, it is useful to apply silane modifications. Many studies have shown improved interfacial adhesion and increased compatibility between starch and the hydrophobic matrix [32,40,44,45]. Moreover, there is less tendency to form agglomerates inside the matrix, and a definite improvement in starch hydrophobicity has been reported. Wei et al. [46] obtained silanized starches with a water contact angle of about 119°, which suggests an enhanced surface hydrophobicity of such starch. Meanwhile, Wang et al. [42] achieved esterified starch/natural rubber composites resulting in increased tensile strength and other mechanical properties. Modifications of starch to increase its hydrophobicity are illustrated in Figure 1 and are discussed in detail in the following publications [32,36,38,42,43,47].

To achieve properties competitive with traditional NR composites, starches must be subjected to modification. In rubber technology, the silanization of fillers is one of the most optimal, effective, and impactful modification methods for the final properties of rubber composites [48]. The significant challenge in reinforcing composites with fillers such as silica or starch (which contain hydroxyl groups) is, as previously mentioned, their tendency to absorb water and their tendency to agglomerate, leading to larger particle sizes. This results in poorer reinforcement compared to carbon black incorporation and a reduction in the hydrophobicity of the composite which, for applications such as coatings, is an important parameter. Consequently, researchers have begun to investigate various coupling agents, particularly silanes, for their potential benefits. In 1988, Nasir et al. [49] studied the effects of silane coupling agents on silica-filled elastomers. They found that reinforcing interfacial interactions occur through condensation reactions between alkoxyorganosilane groups and hydroxyl groups on the surface of the filler (e.g., silica). This reaction releases aliphatic alcohol as a waste product [50,51]. Such interactions lead to stiffening of the composite, improvement of mechanical properties, and modification of rheological properties due to strong interactions between rubber and filler, not only hydrodynamic effects. The mechanism of reinforcement with silica or starch is mainly related to polymer–filler interactions due to the weakening of filler–filler interactions and better filler dispersion; furthermore, the modification of the filler surface causes an increase in the reinforcement coefficient [51,52]. This was described by Kaewsakul et al. [48] for silica-filled NR in the presence of bis(triethoxysilylpropyl)tetrasulfide (TESPT) as a bifunctional silane coupling agent. The action occurs through a process of silanization or hydrophobization at the mixing stage. TESPT silane interacts with the surface hydroxyl group of silica, reducing its hydrophilic character. Moreover, the TESPT-modified silica bonds to the rubber chains during the vulcanization process. To improve the compatibility between the hydrophobic NR matrix and the hydrophilic silica filler, TESPT acts as a reactive compatibilizer and coupling agent. At the same time, it promotes interfacial adhesion [53,54] and bonds the filler and polymer matrix. Besides the advantages mentioned, the application of silane coupling agents as reinforcing agents still has several significant disadvantages, such as the production of alcohol as a waste product of the coupling reaction, the high cost of silane coupling agents, and the complex processing behavior [48,50,54]. Silanization, compared to other modification methods, has a much greater reinforcing effect, which is why it was used in this study instead of, for example, esterification and etherification. Due to the lack of many publications on the effect of silanized starch on vulcanizates, this type of modification was chosen in the present study because both silica fillers and starch require coupling agents to achieve a reinforcing effect. In addition, silanization results in greater hydrophobicity, which can contribute to better interfacial interactions, reduces moisture absorption by the filler, and can positively affect the vulcanization process by forming additional bonds between the rubber during the cross-linking and preventing reversion [53,55].

Research on starch/natural rubber composites [18,19,56,57,58] supports the claim that the incorporation of an adequate amount of modified starch as a filler can increase tensile strength, improve hardness, and increase tear resistance. Another important aspect is the improvement of swelling resistance and barrier properties of the composites. Further, according to a study [59], modified starch affects the values of the storage modulus. It first decreases and then increases as the modified starch content increases. This is due to the formation of a network between the rubber and starch and the strength of the interaction at the filler–rubber interface. This is evidence that the filler–filler network is strengthened by increasing the loading of modified starch. Modified starch also affects rolling resistance. The internal plasticization of NR by the modified starch, and thus the increase in the activity of NR molecules, improves the elasticity of rubber and reduces the internal friction between starch molecules and rubber molecules.

Investigations of starch as a filler in rubber composites are not limited to starch in crystalline form. Stelsceu et al. [60] used plasticized starch to fill natural rubber. The incorporation of plasticized starch resulted in several significant changes, including an increase in the hardness of the composite. Also, tensile strength increases, the elongation at break decreases, and tensile strength has a heterogeneous variation. The optimum curing time decreases, and the swelling of the composite in water, ethanol, sunflower oil, and 10% glucose solution decreases.

In another study [61], a composite of natural rubber and plasticized starch (PS) was cross-linked with peroxide in the presence of trimethylolpropanetrimethyl trimethacrylate. Plasticized starch in amounts of up to 20 phr was found to have a reinforcing effect on natural rubber.

Plasticized starch was also used in [62] in the presence of precipitated silica and organic montmorillonite (OMMT) as a hybrid reinforcing system in the presence of bis-[3-(triethoxysilyl)-propyl]-tetrasulfanesilane (TEPS). Tests showed changes in vulcanization properties with increasing minimum torque. Improvements in the mechanical properties of the samples were observed with the incorporation of fillers. This is due to the reinforcing effect of the nanofiller; by adding an adhesion promoter between the rubber and the filler, an improvement in tensile strength was obtained.

For the most part, natural rubber/starch compositions reported in the literature [18,19,20,60,61,62,63] have been produced by plasticizing or making a starch paste and then mixing it with an elastomeric matrix or mixing it with latex and coagulation. These methods need greater precision and a suitable environment during the chemical reactions. As a result, such procedures require multiple operations on the process flow, with a high consumption of dissolvers and long time required. In contrast, in the present study, starch was silanized using two methods. One by a simple in situ mixing method and the other by condensation reactions from the solution at 50 °C. This resulted in fewer steps during composite preparation. In this paper, the modification of starch by grafting n-octadecyltrimethoxysilane to reduce the hydrophilicity of the filler is described, and the effect of the modified starch on the functional properties and the degree of dispersion in the elastomeric matrix is studied. Natural rubber was used as the matrix, as it is considered a sustainable material, and combined with starch to form a bio-composite that increases the demand for environmental protection. The produced material was characterized by studying cross-linking kinetics, contact angles, mechanical properties before and after thermo-oxidative aging, and dynamic properties. The filler and composite morphologies were analyzed by scanning electron microscopy (SEM), and the attachment of silane groups to the starch was confirmed by Fourier transform infrared spectroscopy (FT-IR). In addition, the surface free energy of starch and starch grafted with silane were determined using the contact angle. Finally, the results of the study were analyzed and summarized, and the effect of starch modification on NR composites was discussed to find potential applications as hydrophobic products.

## 2. Materials and Methods

### 2.1. Materials

To investigate, natural rubber (NR, RSS I) from Torimex-Chemicals Ltd. Sp. z o. o. (Konstantynów Łódzki, Poland) was used as the elastomeric matrix. To cross-link the elastomer, the following components were used: sulfur (S_8_) as a cross-linking agent with a density of 1.8–2.36 g/cm^3^, supplied by Chempur (Piekary Śląskie, Poland); zinc oxide (ZnO) as a cross-linking activator with a density of 5.6 g/cm^3^, obtained from Chempur (Piekary Śląskie, Poland); 2-mercaptobenzothiazole (MBT) as a cross-linking accelerator with a density of 1.29 g/cm^3^, delivered from Sigma-Aldrich (St. Louis, MO, USA); and stearic acid (SA), functioning as a cross-linking activator and dispersing agent with a density of 0.94 g/cm^3^, obtained from Chempur (Piekary Śląskie, Poland). Corn starch with a density of 1.51 g/cm^3^, BET surface area of 1.13 m^2^/g, and purity >99%, sourced from Biomus Ltd. Sp. z o. o. (Lublin, Poland), was used as a filler. For modification of the filler, n-octadecyltrimethoxysilane (OTMS) with a density of 0.88 g/cm^3^ and 85% content of n-isomer, supplied by abcr GmbH (Karlsruhe, Germany), was used.

As solvents to study the degree of cross-linking, the following substances were used: toluene with a density of 0.87 g/cm^3^, delivered by POCh S.A. (Gliwice, Poland); hexane with a density of 0.66 g/cm^3^, sourced by POCh S.A. (Gliwice, Poland); methanol delivered with a density of 0.78 g/cm^3^ by POCh S.A. (Gliwice, Poland); and diethyl ether with a density of 0.71 g/cm^3^, obtained from Chempur (Piekary Śląskie, Poland). In addition, the materials used for silanization were ethanol, distilled water, hydrochloric acid, and potassium hydroxide. All reaction reagents were sourced by POCh S.A. (Gliwice, Poland).

### 2.2. Silanization of Corn Starch

**Figure 2:**
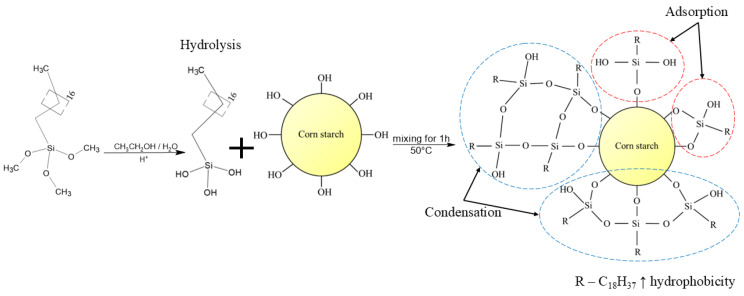
Scheme of corn starch silanization process.

The starch modification was carried out by physical adsorption, chemical grafting, and condensation reactions. Figure 2 shows a scheme of starch silanization for OTMS grafting.

Starch silanization was performed in a three-neck flask using a 300 mL solution of water and ethanol (ratio 1:4 *v*/*v*). OTMS was added and pre-hydrolyzed while stirring for 1 h and maintaining pH = 4–5 with hydrochloric acid. Then, corn starch (30 g) was added, and the temperature was kept at 50 °C while stirring for another hour. The weight ratio of silane/starch was 1:10. After the reaction, the solution was neutralized with potassium hydroxide and cooled to room temperature. Next, the mixture was filtered under reduced pressure, and the precipitate was dried in an oven for 24 h at 60 °C.

### 2.3. Characterization of Chemical Structure of Modified Starch with Fourier Transform Infrared Spectroscopy (FT-IR)

Differences in the chemical structure of modified and unmodified starch were characterized by Fourier transform infrared spectroscopy (Thermo Electron, Nicolet 6700 spectrophotometer, Waltham, MA, USA), and 64 scans were taken between 4000 and 500 cm^−1^ with a resolution of 4 cm^−1^. A single reflection diamond ATR crystal on a ZnSe plate and DTGS/KBr detector were applied. Both starches before measurement were dried for 24 h at 60 °C.

### 2.4. Determination of Surface Properties

To determine contact angles and surface free energy, the starch was pressed into disks under pressure using a hydraulic press, while for NR/starch composites, ~1 mm thick samples were used. The entire procedure below was applied to both the filler and to the composites. The static contact angle between a drop of water, diiodomethane, or ethyl glycol (1 µL) and the compressed starch disk or NR sample was measured using the settled drop method on a goniometer (DataPhysics, OCA 15EC, San Jose, CA, USA). Surface free energies were then determined using SCA 20 software for analysis of the results and calculations. At least five results were obtained for each prepared sample. The Owens–Wendt–Rabel–Kaelble (OWRK) method was used to determine the SFE and calculate the dispersive and polar components of the surface free energy using the following Equations (1)–(3):

(1) $$\gamma_{s}=\gamma_{s}^{P}+\gamma_{s}^{D}$$

(2) $${\left(\gamma_{s}^{P}\right)}^{0.5}=\frac{\gamma_{w}\times (\cos\theta_{w}+1)-2\times 2\times \sqrt{\gamma_{w}^{D}-\gamma_{s}^{D}}}{2\times \sqrt{\gamma_{w}^{P}}}$$

(3) $${\left(\gamma_{s}^{D}\right)}^{0.5}=\frac{\gamma_{D}\times (\cos\theta_{D}+1)-\sqrt{\frac{\gamma_{D}^{P}}{\gamma_{w}^{P}}\times \gamma_{w}\times (\cos\theta_{w}+1)}}{2\times \left[\sqrt{\gamma_{D}^{D}-\sqrt{\gamma_{D}^{P}\times \frac{\gamma_{w}^{D}}{\gamma_{w}^{P}}}}\right]}$$

where γ_s_—total surface free energy of sample (mJ/m^2^); γ_s_^P^—polar component of surface free energy surface (mJ/m^2^); γ_s_^D^—dispersive component of surface free energy (mJ/m^2^); θ—contact angle (°); γ_w_—total free surface energy of water (72.6 mJ/m^2^); γ_w_^P^ and γ_w_^D^—polar (51.0) and dispersive (21.6) components of surface free energy of water (mJ/m^2^), respectively; γ_D_—total free surface energy of diiodomethane (50.8 mJ/m^2^); and γ_D_^P^ and γ_D_^D^—polar (2.3) and dispersive (48.5) components of surface free energy of diiodomethane (mJ/m^2^), respectively.

### 2.5. Assessment of Surface Morphology of Corn Starch and NR Composites

The study of vulcanizate and filler morphology was carried out using an inverted scanning electron microscope (SEM), namely a Hitachi TM-1000 tabletop microscope from Tokyo, Japan. Sample preparation consisted of bonding the test sample on a special table using double-sided adhesive film. The gold layer was then applied to the sample using a Cressington Sputter Coater 108 automatic vacuum sputtering device from Redding, CA, USA, at a pressure exceeding 40 mbar for 60 s. The prepared samples were then introduced into the chamber of a scanning electron microscope for analysis.

### 2.6. Compounding and Vulcanization

Natural rubber composites filled with corn starch composites were prepared using a two-roller mill (type: Laborwalzwerk, Krupp-Gruson, Magdeburg-Buckau, Germany) with a roller diameter of 200 mm and a roller length of 450 mm at a roller temperature of 30–35 °C. The total composition time was 5–8 min. First, the rubber was plasticized, and the components were added in the following order: stearic acid, zinc oxide, filler, accelerator, and sulfur. The complete rubber composites were stored separately in foil at room temperature. The produced mixtures were vulcanized in hydraulic presses in suitable metal molds. The vulcanization parameters were a temperature of 160 °C, pressure of 150–180 bar, and curing time of 5 min. The formulations of the NR compositions are summarized in Table 1. The proportions shown in Table 1 were determined based on knowledge obtained from the literature [18,19,42,60]. Studies show that the addition of 10–20 parts by weight of starch yielded the most optimal property for vulcanizates. Moreover, preliminary studies showed that such mixed compositions had good processing and performance properties.

### 2.7. Evaluation of Vulcanization Process of NR Composites

The curing kinetics of NR composites were determined using an Alpha Technologies (MDR 2000) oscillating disc rheometer (Alpha Technologies, Hudson, OH, USA) at 160 °C (ASTM standard D5289-17 [64]), which was used to determine the following parameters: scorch time (t_02_); vulcanization time (t_90_); minimum torque (T_min_); maximum torque (T_max_); torque increment (ΔT), which is the difference between maximum torque and minimum torque; and cure rate index (CRI). Equations (4) and (5) show the formula for torque increment

(4) $$∆T=T_{max}-T_{min}$$

where ΔT—torque increment (dNm), T_max_—maximum torque (dNm), and T_min_—minimum torque (dNm).

(5) $$CRI=\frac{100}{t_{90}-t_{02}}$$

where CRI—cure rate index (min^−1^), t_90_—vulcanization time (min), and t_02_—scorch time (min).

### 2.8. Swelling Properties of Tested NR Vulcanizates

Swelling behavior was assessed using toluene (according to ASTM D471 [65]). From each vulcanizate, four test pieces of 25–60 mg of different shapes were cut out; weighed using an analytical balance; and next swollen in toluene, methanol, and hexane until equilibrium was reached (for 72 h). After this time, the swollen samples were removed from toluene or hexane and washed with diethyl ether, and their weights were determined again. The samples were dried to a constant weight at a temperature of 50 °C and then reweighed.

Equilibrium volume swelling (Q_v_) was calculated using Equation (6):

(6) $$Q_{v}=Q_{w}\times \frac{d_{v}}{d_{s}}$$

where Q_w_—equilibrium mass swelling (mg/mg), d_v_—vulcanizate density (g/mL), and d_s_—solvent density (g/mL).

Equilibrium weight swelling was calculated from Equation (7):

(7) $$Q_{w}=\frac{m_{s}-m_{d}}{m_{d}^{*}}$$

where m_s_—swollen sample weight (mg), m_d_—dry sample weight (mg), and m_d_^*^—reduced sample weight (mg). The reduced sample weight was calculated from Equation (8):

(8) $$m_{d}^{*}=m_{d}-m_{0}\times \frac{m_{f}}{m_{t}}$$

where m_0_—initial sample weight (mg), m_f_—filler and inorganic part weight in the sample (mg), and m_t_—total sample weight (mg).

The content of the eluted fraction in solvent (−Q_w_), interpreted as the amount of leaching substances, was calculated from Equation (9):

(9) $$-Q_{w}=\frac{m_{0}-m_{d}^{*}}{m_{0}}$$

The rubber volume fraction (V_R_) was calculated from Equation (10):

(10) $$V_{R}=\frac{1}{1+Q_{v}}$$

The degree of cross-linking (α_c_) was determined using Equation (11):

(11) $$\propto_{c}=\frac{1}{Q_{v}}$$

### 2.9. Mechanical Properties Characterization

Properties determined for the vulcanizates were tensile properties, hysteresis loss, Mullins effect, and tear resistance. Tensile strength was evaluated using a testing machine (Zwick1435/Roell GmbH & Co. KG, Ulm, Germany) according to [66].

Hysteresis losses were determined using a testing machine (Zwick1435/Roell GmbH & Co. KG, Ulm, Germany). Each test was performed on three specimens, which were stretched five times to 200% elongation at a tensile speed of 500 mm/min, and the initial force was 0.1 N. The Mullins effect was determined according to Equation (12):

(12) $$E_{M}=\frac{W_{1}-W_{5}}{W_{1}}$$

where W_1_—work required to stretch the sample in the 1st cycle (Nmm) and W_5_—work required to stretch the sample in the 5th cycle (Nmm).

The tear strength (T_s_) was assessed according to method A outlined in ISO 34-1:2015 [67].

The hardness (HA) was measured with a ZwickRoell (Ulm, Germany) hardness tester at ISO 48-4:2018 standard [68].

### 2.10. Resistance to Thermo-Oxidative Aging

The vulcanizates filled with starch underwent thermo-oxidating aging in a forced circulating aging oven maintained at 70 °C for 7 days. Following a conditioning period of 24 h at room temperature, alterations in mechanical properties, including stress at 100% strain (S′_E100_), tensile strength (TS′_b_), and elongation at break (E′_b_), were assessed using the aging factor (AF) according to Equation (13):

(13) $$AF=\frac{{TS}_{b}^{\prime }\times E_{b}^{\prime }}{{TS}_{b}\times E_{b}}$$

where TS′_b_—tensile strength after thermo-oxidative aging (MPa), TS_b_—tensile strength before thermo-oxidative aging (MPa), E′_b_—elongation at break after thermo-oxidative aging (%), and E_b_—elongation at break before thermo-oxidative aging (%).

### 2.11. Determination of Dynamic Properties and Payne Effect

Dynamic properties, including the Payne effect (ΔG), storage modulus (G′), and loss modulus (G″), as well as the tangent of the loss angle (tan(δ)), were determined using an Ares G2 rheometer (New Castle, DE, USA). Disc-shaped vulcanizates with a thickness of ~2 mm were used for the measurement. The sample was then placed between the measuring plates of the device, and the clamping force was 10 N. The measurement was carried out at room temperature. The Payne effect was calculated using Equation (14):

(14) $$∆G=G_{max}^{\prime }-G_{min}^{\prime }$$

where ΔG—Payne effect, G′_min_—minimum storage modulus, and G′_max_—maximum storage modulus.

### 2.12. Procedure of Investigation

**Figure 3:**
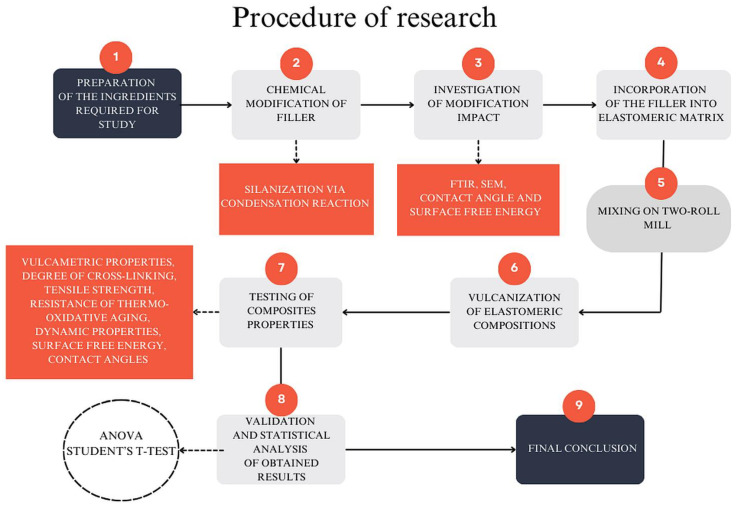
Flowchart of the order of the testing procedure.

The study was divided into two stages. The first stage involved the characterization of the silanization of starch and examining their properties. In the next stage, the impact of silanized starch on composites made of natural rubber was assessed. Figure 3 illustrates what the procedure for testing elastomeric compositions looked like.

### 2.13. Statistical Analysis

To compare the differences between the tested vulcanizates, one-way analysis of variance (ANOVA) was used with the hypothesis that all tested samples were not statistically different from each other. Normal distribution of the results of the tested samples was also assumed, and a confidence interval of 0.95 was used. Moreover, the verified null hypothesis can be presented as follows: H_0_: μ = μ_0_ against the alternatives H_1_: μ ≠ μ_0_, or H_1_: μ > μ_0_, or H_1_: μ < μ_0_. The *p*-value was determined in the program because the analysis was compared with the established significance level (α): if *p* ≤ α—reject H_0_ and accept H_1_, but if *p* > α—there are no grounds for rejecting. During the analysis, equal variances were assumed, and Tukey’s test was used to determine which specific composites’ properties differed from each other. Designations with the same letters in the superscript indicated statistically homogeneous subsets. An independent *t*-test analysis (student’s *t*-test) was used to determine the statistical difference between the silanized starch and pure starch; chemical-modified samples and in situ-modified, assuming equal variances; and compare the differences between aged and unaged composites. A confidence interval of 0.95 was used, so *t* > (t_(*n*−1;α)_), *p* < 0.05 indicates a significant difference between the two samples. Statistics parameters were compiled using the free statistical program Jamovi (version 2.5, Sydney, Australia).

## 3. Results and Their Discussion

### 3.1. Characterization of Modified and Unmodified Corn Starch

#### 3.1.1. Analysis of the Chemical Structure of Starch Before and After Modification

**Figure 4:**
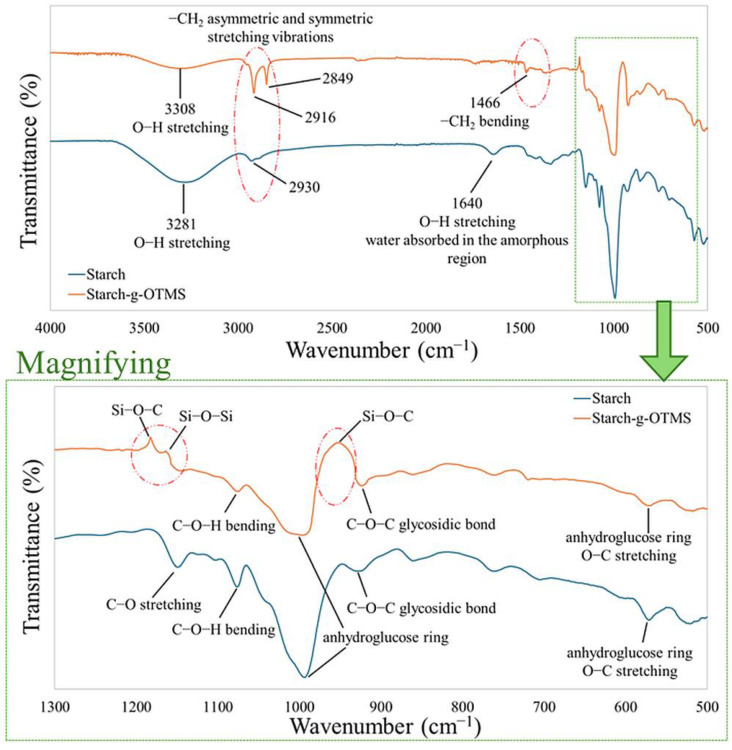
FT-IR spectra of modified and unmodified starch.

Fourier transform infrared spectroscopy (FT-IR) is widely used in polymer analysis because it provides valuable information on the chemical structure and composition of polymers. The method detects specific vibrations of molecular bonds in a polymer, enabling the identification of functional groups [69,70]. In this study, it is particularly useful because of its ability to detect the attachment of new functional groups to starch and confirm that the reaction has occurred. Figure 4 shows two spectrums, one of native corn starch and the other of OTMS-modified corn starch. Moreover, a detailed interpretation of the absorbance bands corresponding to the vibrations of characteristic functional groups occurring in the examined corn starch structures is presented in Table 2.

Unmodified starch indicated that a typical peak with an absorption band at 3281 cm^−1^ was detected, indicating stretching vibrations of -O-H bonds of hydroxyl groups in glucose units [71,72]. For modified starch, a signal from OH groups at 3308 cm^−1^ could also be observed; however, it was less intense, indicating fewer hydroxyl groups and substitution with silane groups and hydrocarbon chains [46]. Next, the peak at 2930 cm^−1^ indicated C-H stretching for NCS. It is worth noting that the spectrum of CS/OTMS in this field had two consecutive peaks at 2916 cm^−1^ and 2849 cm^−1^, indicating an enhanced C-H signal [73,74,75]. The range from 2800 to 3000 cm^−1^ represents asymmetric and symmetric stretching vibrations of aliphatic CH_2_ groups. The spectrum should rearrange four peaks in this band, but practically presented two. This is related to overlapping signals. N-octadeyclotrimethoxysilane has a long aliphatic chain, and after modification and grafting it with starch, changes were observed relative to the spectrum of unmodified starch. This is evidence that the silane, having a long hydrocarbon chain in the structure, was attached successfully. For the spectrum of modified starch, a peak of 1466 cm^−1^ was still noted, corresponding to methyl bending. The peak at 1640 cm^−1^ in the spectrum of unmodified starch was associated with water adsorbed in the amorphous region of starch; after OTMS modification, this peak was flattened and practically invisible [73,74]. It may also be noted that in the FTIR spectra of the hydrophobically modified starch, new peaks close to 1189 cm^−1^ and 1168 cm^−1^ were detected. These new peaks can be attributed to the Si-O-C and Si-O-Si bonds, which resulted from the condensation reaction of hydroxyl groups between OTMS and corn starch [76,77,78]. This is evidenced by the covalent incorporation of OTMS. The signal near 954 cm^−1^ occurring only in modified starch was also due to the vibration of the Si-O-C bond. Other peaks at 1149 cm^−1^ and 1076 cm^−1^ occurred in the spectrum of unmodified corn starch and were responsible for the C-O stretching and C-O-H bending bands, respectively [79,80,81]. Moreover, the 1075 cm^−1^ peak was also responsible for C-O-H binding for hydrophobically modified starch, but its intensity was lower relative to pure starch. Therefore, there was other evidence of substituting hydroxyl groups through silane groups. The other peaks at 993 cm^−1^, 954 cm^−1^, 571 cm^−1^, and 570 cm^−1^ were responsible for the stretching vibrations of the anhydroglucose ring [82,83]. Signals for the 926 cm^−1^ and 924 cm^−1^ peaks, which were due to σ-1,4 glycosidic bonds (C-O-C), appeared for both starch spectra [72]. An acid environment can tear the glycosidic bond; however, at pH = 4–5, the bond is quite stable.

#### 3.1.2. Assessment of the Corn Starch Morphology Before and After Silanization

**Figure 5:**
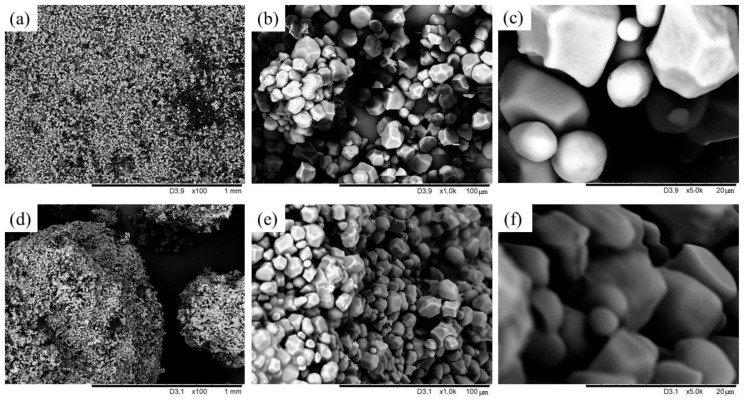
SEM images of native corn starch (**a**–**c**) and silanized starch (**d**–**f**) at different magnifications: (**a**,**d**) 100×, (**b**,**e**) 1000×, (**c**,**f**) 5000×.

The study of the structure and morphology of materials is an important aspect of materials science. It involves understanding related composition and chemistry with processing, micro- or nano-structure, and properties. SEM images are two-dimensional images of surfaces or cross-sections of single- or multi-phase materials. They allow for the assessment of phenomena such as the occurrence of pores or phases, morphology of crystalline grains, and the nature between grain boundaries, but also at phase boundaries in two- or multiphase systems [84,85]. Figure 5 shows SEM images at different magnifications of native corn starch (NCS) and starch modified with n-octadecyltrimethoxysilane (CS/OTMS). At first glance, both starch types appeared similar, but after modification, the starch showed a tendency to form larger clusters and agglomerates of particles. This suggests an improved interaction between molecules, possibly related to the OTMS condensation reaction on the starch surface. Native corn starch particles appeared relatively smooth and irregular in shape, with a more loosely packed arrangement. In contrast, the modified starch particles exhibited tighter packing and more regular shapes. It is not possible to clearly assess the changes in roughness before and after silanization on the images. Unmodified starch particles were slightly larger and irregularly shaped with fewer distinct edges, which is typical of unmodified starch granules. However, modified starch particles appeared more defined and may exhibit slight agglomeration due to the silane modification. This effect could result from improved inter-particle interactions after silanization. When considering starch as a filler, silane modification may offer better interfacial bonding with hydrophobic polymer matrices due to increased compatibility, as silanization introduces functional groups that can bond with polymers. This modification can lead to improved mechanical properties, such as tensile strength and durability, in polymer composites. In contrast, unmodified starch is more hydrophilic and may be less compatible with hydrophobic polymers, which can reduce interfacial adhesion in composites. In summary, silanization defined particle surfaces with potentially enhanced interfacial bonding capacity, improving compatibility and dispersion in hydrophobic materials like natural rubber. This improved interface can lead to enhanced mechanical properties in composites that use silane-modified starch as a filler.

#### 3.1.3. Hydrophobicity and Surface Free Energy of Native and Silanized Corn Starch

Measurement of the contact angle and characterization of the surface free energy of fillers is crucial in the rubber industry, as these parameters directly affect the interfacial adhesion between the filler and the rubber matrix. Good adhesion is essential for reinforcing rubber materials and improving mechanical properties such as strength, durability, and flexibility. The contact angle gives insight into the nature of the filler and, in the case of this study, will allow us to predict whether the modification increased the hydrophobicity of the filler, while the surface free energy will predict the behavior of the filler relative to the elastomeric matrix [86]. Surface free energy is caused by unbalanced forces between the surface and the solid. Total surface free energy is the result of non-polar (dispersive) forces and polar forces and is therefore sensitive to the chemical composition of the surface [87,88].

**Figure 6:**
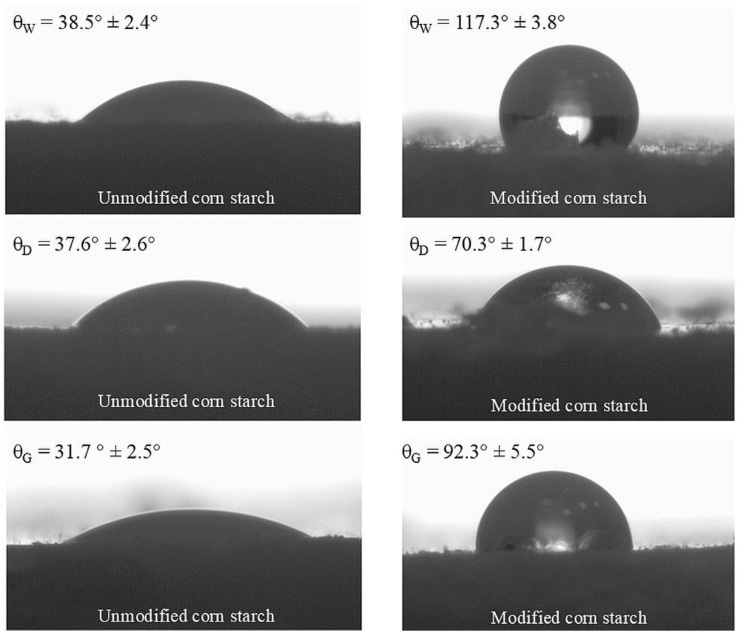
Profile of water droplet, diiodomethane, and glycol ethylene on NCS and CS/OTMS pellets (θ_W_—water contact angle; θ_D_—diiodomethane contact angle; θ_G_—glycol ethylene contact angle).

Figure 6 shows the profiles of droplets settled on the surface of pellets made from native corn starch (NCS) and starch hydrophobically modified by OTMS (CS/OTMS). After analyzing the images, water has a higher affinity for the surface of native starch. The contact angle with water (θ_w_ = 38.5°) confirmed the highly hydrophilic nature of the surface, which is related to the large amount of hydroxyl bonds on the surface, which can form hydrogen bonds with water as affinity increases. For CS/OTMS, significant changes in the character of the surface can be seen. The water droplet had a low affinity for the surface and did not wet the surface. The water contact angle for modified starch was 117.3°, confirming the hydrophobic nature of the sample. This is an indication that most of the hydroxyl groups had been replaced by OTMS with long hydrocarbon chains, which form a brush structure on the starch surface, cutting it off from contact with water. These results confirm the change in the chemical structure of the starch after modification. For the other values of contact angles, that is, for diiodomethane and ethylene glycol, their values increased strongly after modification, which also confirms that modification increased in the hydrophobicity of starch.

A *t*-test was conducted to investigate whether starch modification yielded statistically significant results. Table 3 shows the results of the *t*-test statistical analysis. This analysis was carried out to test statistically significant differences between the contact angle values of two types of starch (native and silanized). The results confirmed that the modification yielded statistically significant differences in the results of the contact angle values.

**Figure 7:**
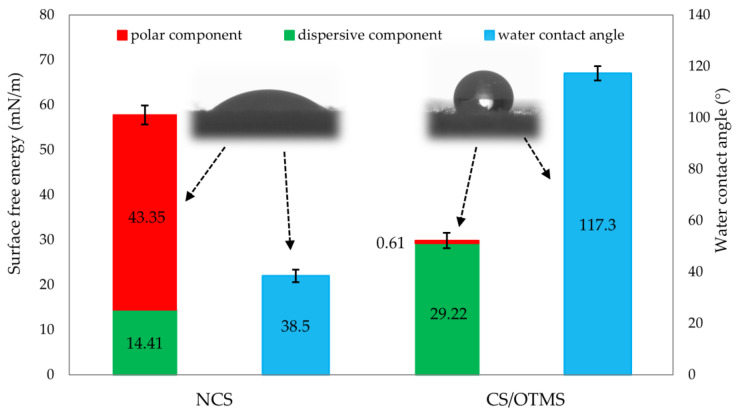
Impact of modification on surface free energy and water contact angle.

Proceeding to the surface free energy (SFE), attention should be paid to Figure 7. It shows the values of water contact angle and surface energy; it is noted that these values were inversely proportional to each other. A large water contact angle value resulted in a smaller SFE and vice versa. This is because SFE includes dispersive and polar components. Greater hydrophobicity will result in lower values of polar forces, resulting in lower values of total surface energy. The values of the dispersion component for unmodified and modified starch were different in relation to the polarity of the substance under study. A change also occurred in the value of the polar component, which, through the substitution of hydroxyl groups by OTMS, decreased due to the inability to form permanent dipoles. CS/OTMS had a marginal polar component, and all of the SFE was responsible for the dispersion component, as evidenced by grafting hydrocarbon chains onto the starch surface. This implies that the hydroxyl groups of the CS surface were substituted by OTMS, and the long chain hydrocarbon is believed to have served as a brush-like structure from the starch surface outward, cutting it off from water. Thus, the high surface free energy value for native starch (γ_s_ = 57.75 mN/m) was associated with strong interactions of hydroxyl groups, which formed stable dipoles. When such starch was added to the elastomeric (hydrophobic) matrices, it was difficult to obtain a well-dispersed morphology. In contrast, modified starch has a much lower surface free energy value (γ_s_ = 29.82 mN/m), including a negligible polar component. Such modified starch allows for obtaining a well-dispersed composite structure.

### 3.2. Characteristics of NR Composites Filled with Modified and Unmodified Starch

#### 3.2.1. Determination Morphology by SEM of NR Composites Filled with Corn Starch

**Figure 8:**
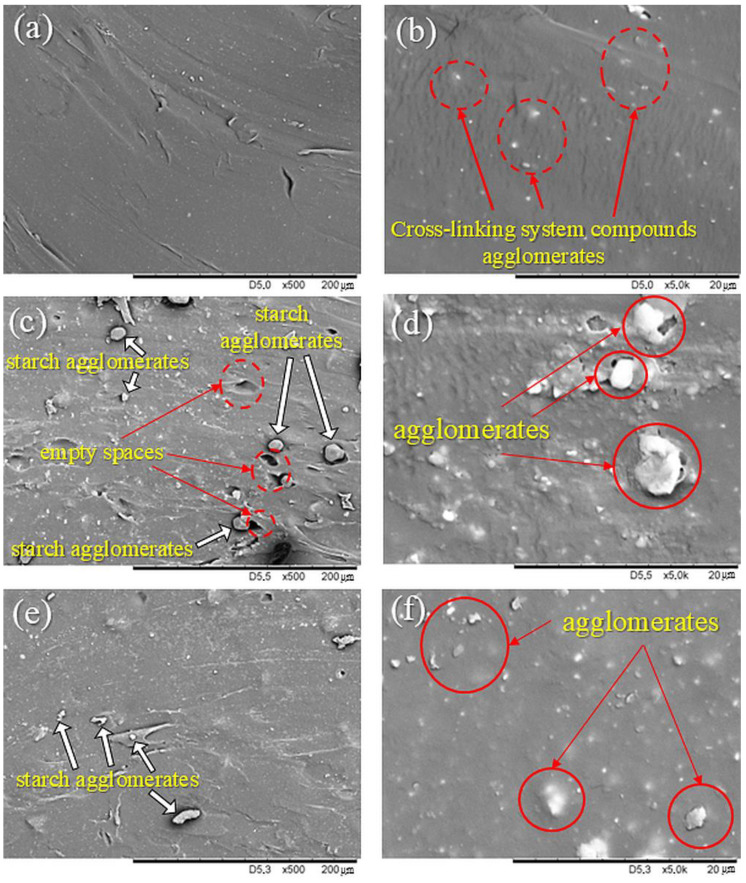
SEM images of natural rubber composites: (**a**,**b**) natural rubber without corn starch, (**c**,**d**) containing 15 phr of native corn starch, and (**e**,**f**) silanized corn starch ((**a**,**c**,**e**)—magnification 500×; (**b**,**d**,**f**)—magnification 5000×).

Scanning electron microscopy analysis was conducted to determine the impact of starch modification on adhesion at the natural rubber–starch phase boundary. This is crucial for understanding the mechanical and dynamic properties of the tested NR composites. Figure 8 shows SEM images of the reference sample filled with 15 phr of native corn starch and 15 phr of modified corn starch at different magnifications. Analysis of the SEM images proves more compatibility of chemically silanized starch with the elastomeric matrix than unmodified starch. In the composite images containing unmodified starches, dark dots were noted. This indicated the empty spaces between rubber and starch agglomerates. In Figure 8c,d, a larger number and larger sizes of starch agglomerates were noticed compared to Figure 8e,f, which proves that for the sample with unmodified starch, the filler interacted with the polymer less. However, in the case of composites filled with CS/OTMS, there were fewer agglomerates, smaller starch particles, and no empty spaces. This suggests better interactions between the polymer and filler, better dispersion of the filler, and greater compatibility between both. This structure may have resulted in improved mechanical and dynamic properties and did not reduce the hydrophobicity of the composites. Improving the compatibility between the filler and the rubber is related to the silanization reaction and the formation of hydrophobic groups on the starch surface, like silanol groups bonded with long aliphatic chains. Chemically attached silane particles acted as a link between the ingredients of the composition. Ultimately, this relationship caused an increase in intermolecular interactions and, consequently, an increase in the degree of cross-linking and overall reinforcement of the material. It is worth explaining that the white dots in Figure 8a,b were probably agglomerates of zinc oxide, the accelerator, or sulfur. No starch was added to the NR0 sample, but it did incorporate a cross-linking agent. Due to its weak compatibility with the matrix, white dots can be observed, which represent agglomerates of the cross-linking agent compounds. This is likely zinc oxide because it possesses the most hydrophilic character among the substances.

#### 3.2.2. Analysis of the Degree of Cross-Linking of NR Composites Filled with Corn Starch

Vulcanization is a chemical process used to transform a mix into a vulcanizate that has functional properties. During vulcanization, sulfur or other cross-linking agents form bonds between polymer chains; this phenomenon is associated with heat transfer but also with chemical reactions occurring in the elastomeric medium [89,90]. The process is designed to increase the rubber’s durability, flexibility, resilience, and heat resistance, making it suitable for a wide range of applications, from tires to gaskets. Without vulcanization, rubber remains sticky and brittle and lacks the durability needed for most practical applications. Therefore, in the study of elastomeric materials, it is crucial to determine the parameters of cross-linking kinetics. These data largely allow for defining the degree of cross-linking of given elastomeric compositions. The degree of cross-linking is important because it is crucial to the mechanical properties of rubber. It refers to the number of chemical bonds formed between polymer chains during the vulcanization process. More cross-linking results in a stiffer, more durable material, while a lower degree provides greater flexibility and softness. However, it is the optimization of this parameter that is important when designing rubber materials for various applications. The degree of cross-linking can also be determined by equilibrium swelling. This test also gives information on the number of cross-links between polymer chains, since the cross-linked material does not dissolve but swells. This swelling behavior is an important indicator of the degree of cross-linking of the rubber, with more cross-linked networks limiting the amount of solvent absorbed and resulting in less swelling [91,92].

Table 4 shows both the vulcametric and swelling properties of the NR vulcanizates tested. The analysis of variance showed significant differences between samples with the following parameters: Q_v_^T^ (F = 19.2; *p* = 0.001), −Q_w_^T^ (F = 186; *p* < 0.001), V_R_^T^ (F = 19.6; *p* = 0.001), Q_v_^H^ (F = 62.3; *p* < 0.001), −Q_w_^H^ (F = 274; *p* < 0.001), V_R_^H^ (F = 37.9; *p* < 0.001), Q_v_^M^ (F = 73.5; *p* < 0.001), −Q_w_^M^ (F = 84.8; *p* < 0.001), V_R_^M^ (F = 76.4; *p* < 0.001), and α_c_ (F = 20.2; *p* = 0.001).

**Figure 9:**
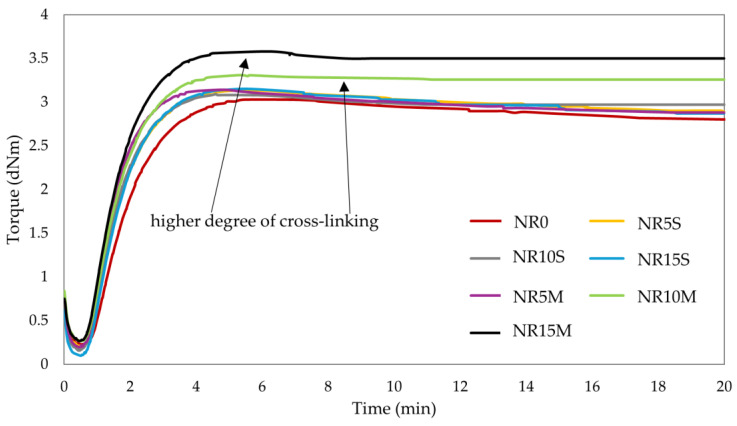
Vulcanization curves for tested NR composites filled with corn starch at T = 160 °C.

The elastomeric compositions were used to study different methods of filler modification. Adding unmodified starch alone to a hydrophobic matrix such as natural rubber did not improve the mechanical, dynamic, or surface parameters due to its hydrophilic character. Therefore, it was decided, in addition to starch modified with OTMS through chemical reactions, we tested how in situ modification of starch with the same compound at the same mass ratio affects the properties of NR composites. Analyzing the results in Table 4, it should be concluded that the addition of starch resulted in a slight reduction in scorch and vulcanization times. However, after considering the results, it should be concluded that starch is not a filler that negatively affects vulcanization time. Both the highest increase in torque and the highest value of maximum torque were shown by sample NR15M (ΔT = 3.31 dNm; T_max_ = 3.58 dNm). Therefore, it should be concluded that it had the highest degree of cross-linking among the tested samples. Notably, the samples with chemically modified starch achieved higher increments and values of torque than their in situ modified starch counterparts. In general, the addition of starch caused NR composites to have higher T_max_ values and a higher degree of cross-linking than unfilled NR vulcanizate. The greater the CS/OTMS addition, the greater the T_max_ and ΔT values, resulting in an increase in the degree of cross-linking (NR5M: ΔT = 2.94 dNm, NR15M: ΔT = 3.31 dNm). Such a trend is not seen in samples where starch with silane was added in situ. It is likely that unbound silane can affect the process of cross-linking and ultimately the vulcametric parameters studied. Figure 9 shows the vulcanization curves of the tested samples. Conventionally cross-linked natural rubber is particularly sensitive to reversion, as can be seen in Figure 9. The phenomenon of reversion is undesirable in rubber materials and results in deterioration of properties, mainly mechanical. This is primarily due to the degradation of the cross-links, which are destroyed by overheating and extended curing. It should be noted that NR vulcanization is a dynamic equilibrium process in which cross-links are constantly formed and broken. If the curing time is too long, the equilibrium shifts toward bond breakdown. The incorporation of starch made it possible to avoid reversion and eliminate this unfavorable phenomenon for rubber products, so this should be noted as a benefit for this filler. The curves confirm the results of torque increments from Table 4 in the case of the degree of cross-linking.

The results of the equilibrium swelling test indicated that the higher the starch content of the samples, the higher the degree of cross-linking (Table 4). Composition NR0 obtained the highest Q_v_ value in both toluene and hexane (5.17 mL/mL and 2.61 mL/mL, respectively), proving a low degree of cross-linking. Samples tested in toluene showed higher Q_v_ values than those determined in hexane. This pointed to the composites tested being more sensitive to solvents with aromatic groups. Composites filled with CS/OTMS obtained lower Q_v_ values in both toluene and hexane than their counterparts with NCS. Such a phenomenon is related to filler–elastomer interactions. When the rubber is not filled, the solvent can afford to infiltrate and penetrate the material, as there are no barriers or limitations. Filled vulcanizate is characterized by a more packed structure, which provides more resistance to the solvent, and penetration is limited so that the material does not swell as much [91]. CS/OTMS activities with the elastomeric matrix were higher than for samples with starch and silane additions, for which toluene and hexane had problems with penetration and swelling of the NR composites tested. The amount of leached fraction (−Q_w_) in both toluene and hexane increased with starch content. However, as in the case of Q_v_, composites with the addition of chemically modified starch obtained smaller values than their counterparts. This indicated the possibility of leaching out some of the filler. In addition, it is worth noting that modified starch, due to its greater hydrophobicity, can dissolve partially in apolar solvents when it does not bind with rubber (NR0: −Q_w_^H^ = 0.101 mg/mg, NR10M: −Q_w_^H^ = 0.169 mg/mg). The volume fraction of rubber in the swollen sample (V_R_) showed an inverse relationship to that described for Q_v_. The presence of filler increased the V_R_ value, while chemical modification with silane raised the V_R_ value relative to the other samples. Samples NR10M and NR15M obtained the highest V_R_ values in toluene and hexane, confirming their highest degree of cross-linking among the samples tested. The equilibrium swelling results confirmed what the cross-linking kinetics study showed. Subliminally, the highest degree of cross-linking was achieved by sample NR15M (α_c_ = 0.235). To complement the swelling studies, this composition was also tested in a polar solvent such as methanol. Due to the incorporation of starch, which, unmodified, is hydrophilic in nature, it was verified whether it affects resistance to polar solvents. The results showed the opposite trend to toluene and hexane. The Q_v_^M^ value increased with the content of both modified and native starch, which may be related to the unbound part of the filler. This phenomenon was probably due to the sorption of methanol by the unbound and unmodified starch particles, by which the sample gains weight and swells. It was also confirmed by the V_R_^M^ result and its decreasing trend with filler content. The amount of leached fraction (−Q_w_^M^) also increased with starch content; however, it is worth noting that for samples with CS/OTMS, it took on smaller values than for native starch. These values suggest better interaction of silanized starch with the elastomeric matrix (NR15S: −Q_w_^M^ = 0.185, NR15M: −Q_w_^M^ = 0.172). In conclusion, the swelling study in methanol, due to the low Q_v_^M^ values, showed little effect of polar solvents on the NR composites tested. The incorporation of both starches increased this parameter; however, the addition of CS/OTMS allowed for achieving a higher α_c_ than the incorporation of the same amount of unmodified starch (dependent on better-dispersed filler and interfacial interactions).

#### 3.2.3. Evaluation of Mechanical Properties and Thermo-Oxidative Aging Resistance of NR Composites Filled with Corn Starch

The mechanical properties of rubber products are crucial in determining their performance in various applications. Rubber’s unique combination of elasticity, flexibility, and strength is due to its molecular structure, allowing it to deform significantly under stress and return to its original shape when the stress is removed. These properties are highly dependent on factors such as the type of rubber, the degree of cross-linking during vulcanization, and the use of additives such as fillers and plasticizers. It is well known that the mechanical properties of cross-linked rubber compositions are improved by the inclusion of fillers. In addition, such enhancements are associated with filler particles (agglomerates) and rubber–filler interactions.

Tensile strength is an important mechanical property of rubber materials. It describes the amount of stress the material can withstand when stretched before it breaks. Thus, it reflects rubber’s ability to withstand the forces that try to destroy it. Analyzing the results, it was found that the incorporation of CS/OTMS resulted in an improvement in mechanical properties relative to the reference sample. In contrast, the incorporation of NCS in the presence of silane worsened the tensile strength. This was probably caused by the formation of stress inside the matrix due to the formation of starch agglomerates and their hydrophilic nature.

Composites with the addition of CS/OTMS increased the tensile strength parameter, as sample NR5M showed a tensile strength that was 0.4 MPa higher (11.0 MPa). In contrast, samples NR10M and NR15M achieved the highest tensile strength, TS = 11.4 MPa. That is, with a tensile strength of 0.8 MPa more than NR0, there was a 7.5% increase over the reference sample. The tensile strength results clearly show that CS/OTMS interacted better with the elastomeric matrix to form a reinforcing network in the composite, allowing it to carry loads in a better way. The incorporation of the chemically modified filler does not cause the sample to stiffen as evidenced by the stress values at 100%, 200%, and 300% stretch. For comparison, S_E100_ = 0.560 MPa for NR0, while for NR15M, S_E100_ = 0.668 MPa, which is a change of about 20%. Adding CS/OTMS both stiffened the vulcanizate more and gave it greater strength than adding starch without chemical modification (for example, for NR10S and NR10M, S_E100_—0.574 MPa and 0.633 MPa, respectively, and TS_b_ = 9.8 MPa and 11.4 MPa, respectively). Each of the samples tested achieved elongation greater than 1200%, the maximum that could be measured on the available apparatus. Adding starch did not change elongation, which is a positive aspect in the context of flexible products. Table 5 summarizes the tensile strength results before and after aging for the NR vulcanizates tested. The analysis of variance showed statistically significant differences for the given strength parameters: S_E100_ (F = 135; *p* < 0.001), S′_E100_ (F = 96.8; *p* < 0.001), TS_b_ (F = 11.7; *p* < 0.001), TS′_b_ (F = 29.4; *p* < 0.001), and AF (F = 22.1; *p* < 0.001). Relative elongation before and after aging was not considered due to the values close to the limit of possibilities in this parameter in the strength machine.

Thermo-oxidative aging refers to the degradation process that occurs in rubber when it is exposed to elevated temperatures and oxygen. This combination of heat and oxygen causes chemical reactions in the rubber, leading to changes in its physical and mechanical properties, often resulting in a loss of elasticity, weakened tensile strength, brittleness, and surface cracking of the rubber. The aspect of aging resistance is crucial for rubber products, as it helps determine whether a product will retain its properties when exposed to environmental factors [93,94].

**Figure 10:**
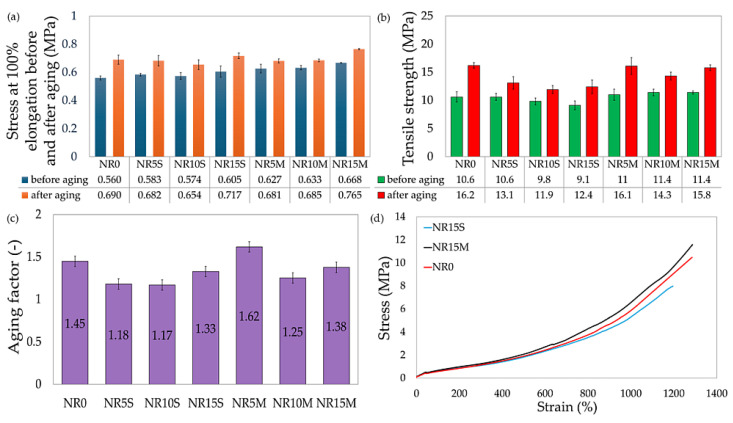
Comparison of mechanical properties before and after aging of the tested NR composites (cross-linked at T = 160 °C for t = 5 min): (**a**) stress at 100% elongation; (**b**) tensile strength; (**c**) aging factor; (**d**) stress–strain chart for NR0, NR15S, and NR15M.

Table 5 summarizes the strength results after aging and before aging, as well as the aging factor calculated from equation (12). The larger the AF value, the more resistant the vulcanizate is to aging. The results suggest that starch does not favorably affect aging resistance, as only one NR5M sample (1.55) obtained a higher AF value than the reference sample. The remaining compositions obtained values less than 1.45. The tensile strength after aging increased for each tested vulcanizate relative to the strength before aging (for NR10S from 9.8 MPa to 11.9 MPa), so the aging factor was more than 1 for each sample. The results in Table 5 show that the CS/OTMS-surfaced samples were more resistant to aging than their counterparts with the same amount of starch added. The values of tensile strength and stress at 100% elongation increased after aging (for NR15M from 0.668 MPa to 0.765 MPa), indicating that the system was cross-linked, and the polymer chain stiffened due to temperature and the presence of oxygen. It should be noted that the composites, despite the increase in stiffness, did not lose relative elongation, as they all gained more than 1200% elongation despite aging. Therefore, it can be concluded that the addition of up to 15 phr of CS/OTMS did not cause a significant deterioration in aging resistance relative to NR0. The situation was different for the NR5S, NR10S, and NR15S samples. A trend was observed that higher amounts of starch resulted in a decrease in aging resistance. Therefore, it should be considered whether the lack of chemical modification of starch affects its behavior in the elastomeric matrix after thermo-oxidative aging. Starch is a highly biodegradable polymer with an inherently hydrophilic nature, so without appropriate surface modification after exposure to oxygen and moisture temperatures, it can cause a reduction in the mechanical resistance of composites. Figure 10 illustrates stress at 100% elongation; tensile strength before and after aging; the aging factor for all tested specimens; and a stress–strain chart for the NR0, NR15S, and NR15M specimens. This choice was made to show the extreme results of this study, where one can see the difference in the filler modification method on the tensile strength result and compare it to the reference sample.

#### 3.2.4. Other Mechanical Properties of NR Composites Filled with Corn Starch

Tear resistance (T_R_) is a characteristic property that measures a material’s resistance to the propagation of cuts or tears once they are initiated. It indicates how well the rubber can withstand further damage when subjected to tearing forces. Table 6 shows the results of the tear strength test. The highest strength was achieved by the reference sample, which was not much of a surprise, due to the high sensitivity to the presence of filler clusters of this parameter. The larger the aggregates, the more sensitive a material is to tearing. The addition of starch did not positively influence this parameter, reducing its value from 6.96 N/mm to 4.47 N/mm. Higher amounts of filler caused decreased T_R_ value however, there was a trend among the filled samples that composites with CS/OTMS along with the amount of added starch had better strength than their counterparts with unmodified starch except NR5S, which showed the highest tear resistance among the filled composites. For the CS/OTMS-filled samples, it was the NR10M sample that achieved the highest tear strength T_R_ = 6.19 N/mm. In this study, there was no such trend in the tensile strength that the more CS/OTMS, the better the mechanical properties.

**Figure 11:**
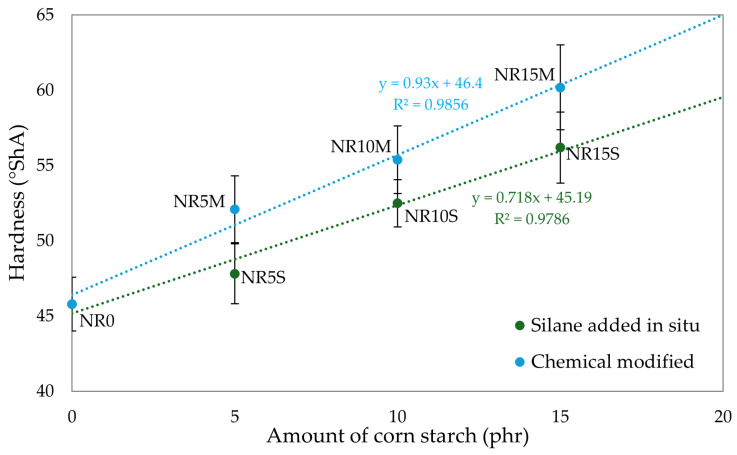
Effect of corn starch amount and modification on the hardness of NR composites (cross-linked at T = 160 °C for t = 5 min).

The effect of added filler on the hardness of the rubber composite is illustrated in Figure 11. The greater the amount of filler, the greater the hardness of the composite due to an increase in cross-linking density but also intensive polymer–filler interactions. The highest hardness was achieved by NR15M vulcanizate (HA = 60.2 °ShA), and the lowest by NR0 vulcanizate (HA = 45.8 °ShA). It is noteworthy that the incorporation of the same amount of CS/OTMS as regular starch in the presence of silane into the NR matrix caused an increase in hardness in some cases of almost 10% (from 47.8 to 52.1 for composites with 5 phr of filler). The results prove that chemically modified starch allows for better interactions at the interface between the filler and matrix, which is a key aspect in the mechanical properties of elastomeric composites (Table 6 and Figure 11). The ANOVA showed statistically significant differences in the studied parameters, such as T_R_ (F = 63.3; *p* < 0.001), HA (F = 41.0; *p* < 0.001), ΔW_1_ (F = 25.4; *p* = 0.002), ΔW_5_ (F = 5.52; *p* = 0.042), and E_M_ (F = 30.1; *p* = 0.001).

Hysteresis loss testing refers to the loss of energy that occurs when a rubber material is subjected to cyclic tension and relaxation. This energy loss is represented by the hysteresis loop on the stress–strain curve. During tension, the rubber absorbs energy, and upon unloading, not all the stored energy is recovered, resulting in a difference between the stress–strain and relaxation curves [95]. The area in the hysteresis loop represents energy lost as heat. It is important to understand rubber’s damping and heat-generating properties, which are crucial for applications requiring energy absorption or insulation. In addition to the hysteresis loop, it is important to calculate the Mullins effect. It describes the behavior of a filled rubber material involving stress relaxation over several stretching cycles. This phenomenon occurs due to the breakdown of the filler network and internal structural changes in the rubber composite during the initial stretching cycle. This parameter is important because it defines how a given elastomeric composite will behave in the long term [96].

**Figure 12:**
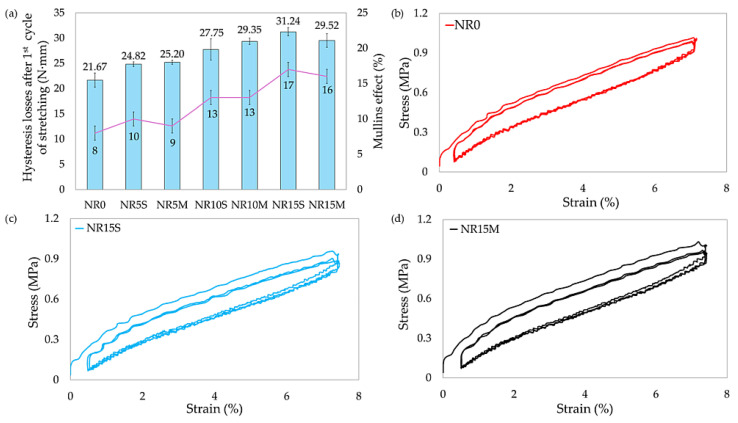
(**a**) Comparison of hysteresis loss values during first stretching cycle and Mullins effect values of tested NR samples, (**b**) NR0 stress–strain hysteresis chart, (**c**) NR15S stress–strain hysteresis chart, (**d**) NR15M stress–strain hysteresis chart (cross-linked at T = 160 °C for t = 5 min).

The data presented in Table 6 show that the starch-filled samples exhibited higher mechanical energy losses, which were greater than those of the reference sample. This phenomenon may be due to the storage of energy inside the rubber material and the dissipation of stresses as thermal energy emission. According to one study [97,98], part of the polymer chains bound to the filler is divided into an induced anisotropic elastic network and two anisotropic damage networks. Among this damage network, one is irreversible and is responsible for the Mullins effect, and the other part is reversible and affects hysteresis. In the studied composites, the more filler, the more networks causing irreversible damage, as confirmed by the E_M_ values (NR0: E_M_ = 8%, NR15S: E_M_ = 17%). In the case of the Mullins effect, which is related to stress softening during the deformation, CS-filled composites showed a higher Mullins effect compared to the reference sample. In the case of filled samples, the modified starch did not significantly change the value of the Mullins effect relative to the addition of unmodified starch in the presence of silane. Larger E_M_ values in compositions with higher amounts of filler are related to the breakdown of the filler network and interactions on the rubber–filler line. This phenomenon is related to the fact that aggregates and agglomerates of the filler can be formed in the elastomeric matrix, which are treated as rigid particles without elastic properties. They interfere with the process of returning to the original shape of the rubber, as part of the energy is dissipated in the form of heat [99]. It is important to note that the loss of hysteresis after one cycle differs to a much greater extent than after five cycles; this is related to the relaxation stresses of rubber composites. Figure 12 presents hysteresis loss values during the first stretching cycle, Mullins effect values of tested NR samples, and stress–strain hysteresis charts of selected specimens. It should be noted that in the stress–strain hysteresis graphs, the reference sample according to Figure 12a had the lowest hysteresis loss value, while the remaining samples showed higher values, which increased with the amount of filling. This phenomenon is related to the disruption of the filler network and filler–polymer interactions. The type of starch modification did not affect this parameter.

#### 3.2.5. Dynamic Properties and Payne Effect of NR Composites Filled with Corn Starch

Dynamic properties are determined using the storage modulus, which measures the recoverable deformation energy in a deformed sample and is responsible for the elastic behavior of the elastomeric composite. The loss modulus, on the other hand, corresponds to the viscous behavior and is a measure of energy loss due to heat. In addition to these parameters, the ratio of the loss modulus to the storage modulus is crucial for rubber composites and is referred to as the loss tangent. This is a measure of damping in the material. The higher the value, the better it dissipates energy, meaning it dampens vibrations more effectively. Lower values of this parameter indicate less energy dissipation and are characteristic of greater elasticity [100]. The optimization of this parameter is important in the context of selecting the application for the composite. Additionally, for rubber products, the Payne effect is significant. It describes the changes in the properties of the elastomeric element as a function of the applied strain. The Payne effect manifests as a dependency of the viscoelastic storage modulus on the amplitude of the applied strain. This effect can be attributed to strain-induced changes in the material’s microstructure. The most accepted model explaining the Payne effect arises from the breakdown of the filler network formed by filler–filler interactions [101]. Table 7 presents the dynamic properties of the tested NR composites.

**Figure 13:**
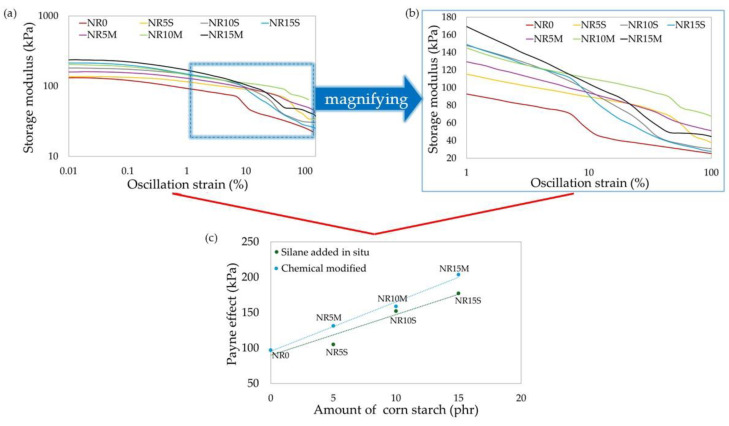
Effect of modified corn starch on dynamic properties of NR composites (cross-linked at T = 160 °C for t = 5 min): (**a**) storage modulus, (**b**) magnifying storage modulus, (**c**) Payne effect.

**Figure 14:**
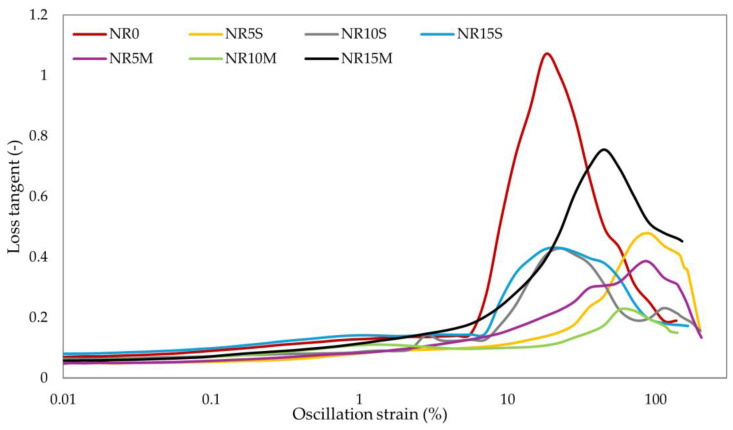
Effect of chemically and in situ silanized starch on tested NR composites loss tangent.

Figure 13 and Table 7 present the storage modulus and Payne effect values for composites containing modified and unmodified corn starch. Modification of the starch significantly affected the activity of the filler, increasing ΔG′. This indicates a significant ability for filler–filler and filler–polymer interactions. The greatest reinforcement effect was obtained for composites containing 15 phr of filler. Moreover, the results confirmed that the most extended spatial network of filler was obtained for composites containing 15 phr of CS/OTMS. An increase in the content of non-chemically modified filler also resulted in an increase in the Payne effect value, however, to a lesser extent compared to the addition of chemically modified starch. As for the results of dynamic properties such as the storage modulus and loss modulus, they are summarized in Table 7. It should be noted that the degree of filling causes an increase in the value of G′, which indicates an increase in elastic interactions and a greater ability to recover and store energy from deformation. The NR15M sample obtained the highest value of G′ equal to 241.1 kPa. The incorporation of CS/OTMS into the rubber resulted in a higher G′ value, which determined a higher energy storage property, greater polymer–filler interactions, and the formation of a spatial network of filler. Figure 14 shows a loss tangent for the tested specimens. Regarding the loss tangent, the NR0 sample obtained a value of 1.07, which suggests a balance between takeoff and energy storage from deformation. The other samples had values less than 1, which suggests more elastic properties of the tested composites and more energy storage capacity than loss. Among the filled compositions, the NR15M sample had the largest value, which suggests the greatest balance among energy storage vs. energy loss and greater damping potential than the other starch-filled samples (Figure 14). Modification of the starch, in addition to NR15M, decreased the viscosity properties and loss energy dissipation during heating deformation. In Figure 13c, it is worth noting that the Payne effect increased with starch content, and the modification positively affected the filler activities. The storage modulus increased with filler content, and energy loss to heat decreased except for the NR15M specimen. The results of the Payne effect and dynamic properties of the tested elastomeric compositions correlated with the values of hysteresis loss and the Mullins effect. Higher values of the Mullins effect caused the sample to achieve higher values of the Payne effect, which agrees with the literature [100,101] and with the phenomenon of formation of a network of filler–filler and filler–polymer interactions. Both effects are related to deformation and stress softening.

#### 3.2.6. Hydrophobicity, Contact Angles, and Surface Free Energy of NR Composites Filled with Corn Starch

**Figure 15:**
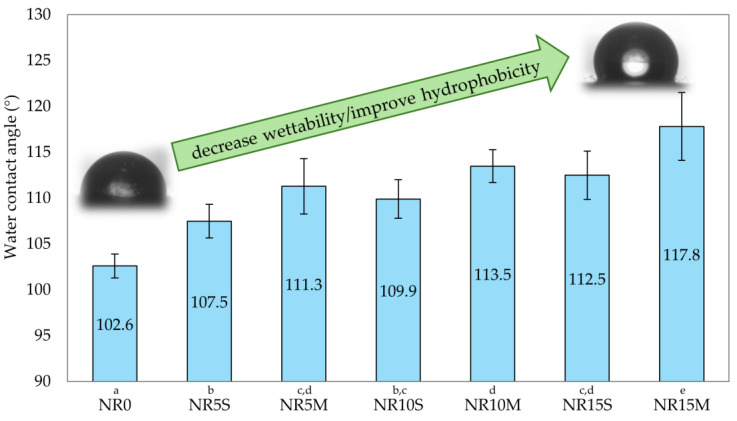
Comparison of water contact angles of the tested NR compositions (cross-linked at T = 160 °C for t = 5 min). Letters above determination of samples indicate statistically homogeneous subsets (Tukey’s HSD test, α = 0.05); ANOVA F = 50.8, *p* < 0.001.

Hydrophobicity is a parameter which is characteristic of seals and membranes. Hydrophobic material should repel water and have low wettability for no substance to adhere to the surface. Because of this situation, such material can be easily kept clean and also does not cause problems with the accumulation of water molecules on the surface. Figure 15 shows the water contact angles of the NR composites tested. The reference sample obtained θ_w_ = 102.6°, which allowed us to conclude that it is a hydrophobic material. However, the addition of a hydrophilic filler such as starch can disrupt the nature of the material. Therefore, it is important to add a modifier or modify by chemical reactions such kind of filler. In this case, both samples with starch added in the presence of silane at a ratio of 1:10 and those with CS/OTMS increased their hydrophobicity with greater filling. The highest water contact angle (117.8°) was achieved by the NR15M sample. The other samples reached values ranging from 107 to 114°. The results clearly showed that CS/OTMS caused a greater effect on the hydrophobic character of the composite than the incorporation of native starch in the presence of silane (NR15M: θ_w_ = 117.8°, NR15S: θ_w_ = 112.5°). It can be concluded that the chemical modification substituted more hydroxyl groups in the starch than the in situ modification, as indicated by the results of the water contact angle of the different compositions.

**Figure 16:**
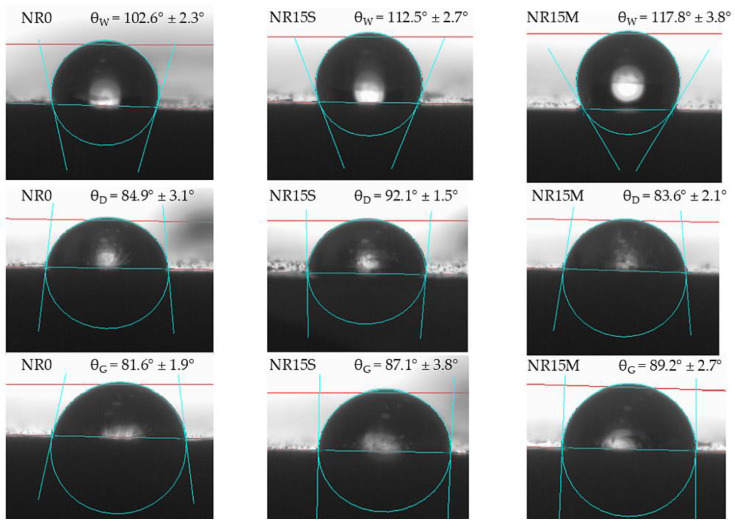
Influence of modified corn starch on the contact angle values of water, diiodomethane, and ethyl glycol (θ_W_—water contact angle; θ_D_—diiodomethane contact angle; θ_G_—glycol ethylene contact angle).

**Figure 17:**
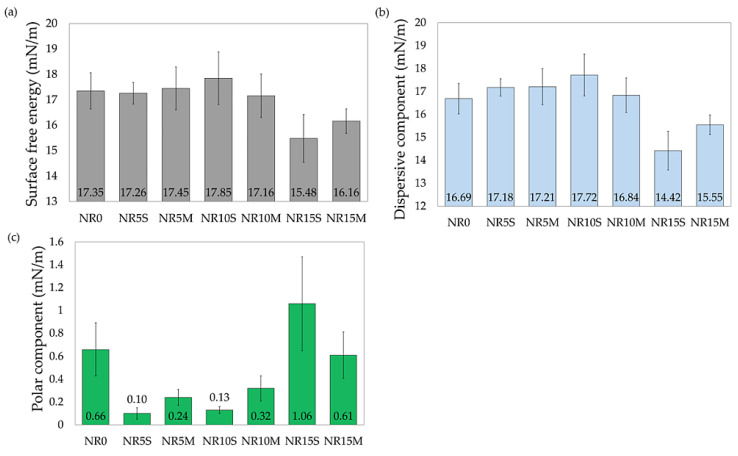
Impact of modified corn starch on surface free energy parameters: (**a**) total, (**b**) dispersive, and (**c**) polar components.

Figure 16 shows a comparison of photos of droplet profiles of three different liquids on the surface of three different samples. For this, the NR0, NR15S, and NR15M vulcanizate were chosen. These samples were chosen because they show noticeable changes in contact angles. Three different liquids allow for the calculation of the surface free energy of a material. This makes it possible to assess intermolecular interactions and compatibility, in this case, between the filler and the elastomeric matrix as well as adhesion to other substances, wettability, and hydrophobicity. In addition, the surface free energy of the NR composites was calculated, which confirms the conclusions of the contact angle study. However, the surface free energy values are very close to each other and are dominated by the dispersion component, indicating the non-polar nature of the surface. The results also indicate low wettability and low adhesion to other surfaces, such as membranes, gaskets, or hydrophobic coatings, which may indicate the use of such composites. The low values of the polar component indicate the NR composite’s resistance to environmental factors such as moisture, as it has a low affinity for polar liquids. Among the compositions tested, the lowest SFE value was obtained by the NR15S sample (15.48 mN/m). Adding the filler caused the SFE in both cases to decrease in surface energy values relative to the reference sample; however, this was not a significant reduction in value. In the context of using a hydrophilic filler such as starch, its addition in the systems used had little effect on the increase in polarity and affinity for polar solvents such as water, as shown by the results in Figure 17. It should be noted that 15 phr is not such a content of starch that could significantly affect the surface free energy values.

#### 3.2.7. Statistical Analysis of Impact Modification on Selected Properties of NR Composites Filled with Corn Starch

The *t*-test is commonly used in materials science to compare the means of two sets of data—often between a treated and an untreated sample or between two different material properties—to determine whether the differences are statistically significant. In materials research, *t*-tests can help verify the effects of additives, assess changes in durability, or understand how modifications affect material performance under different conditions. However, in the case of the present study, the *t*-test was designed to investigate statistically significant differences in the type of starch modification and its effect on the properties of the elastomeric composites tested.

Table 8 shows the differences in the basic parameters describing the elastomeric composites. The statistical analysis carried out showed that for the most part, chemical modification made the parameters change significantly. No statistical differences were observed for such parameters, such as the degree of cross-linking (except for 15 phr of filler) or the Mullins effect (except for 5 phr of filler). This indicates that the type of modification does not significantly affect these parameters. In the samples with 15 phr of filler, the type of modification is key, as all the tabulated parameters showed statistical differences except EM. In conclusion, chemically modified starch has a statistically significant effect on the properties of elastomeric products when it is incorporated into them

## 4. Conclusions

Based on the research, the following conclusions were reached:

1. The effective use of starch as a filler in natural rubber composites was achieved. A plant-based composite was successfully made. The research confirms that starch can be used as a filler; however, it needs chemical modification for better interaction with the elastomeric matrix.
2. FTIR analysis confirms the effective modification of starch with n-octadecyltrimethoxysilane.
3. Silanization significantly impacted the characteristics of starch, rendering it hydrophobic and lowering its surface energy. The water contact angle increased from 38.5° to 117.3°, while the surface free energy decreased to 29.83 mN/m. Additionally, the polar component dropped from 43.35 mN/m to 0.61 mN/m.
4. Silane modification creates smaller but tighter packing starch particles, enhancing their interaction with the rubber matrix and reducing void formation compared to native starch. Analysis of the morphology of starch-filled elastomeric composites showed better filler dispersion after chemical modification by silanization reactions.
5. Incorporation of modified starch influenced the enhanced degree of cross-linking. The NR15M sample obtained α_c_ = 0.235 and T_max_ = 3.58 dNm. The higher the starch content, the higher the degree of cross-linking of the vulcanizates.
6. Composites containing silanized starch demonstrated enhanced mechanical properties, including increased hardness and tensile strength. The hardness increased from 45.8 for NR0 to 60.2 °ShA for NR15M, while the highest tensile strength was obtained for samples NR15M and NR10M with a TS_b_ = 11.4 MPa.
7. Hysteresis testing, related to stress softening, indicated that a higher starch content allowed for greater hysteresis losses, resulting in higher Mullins effect values.
8. The addition of starch reduced the tear strength due to filler agglomerates, while the use of modified starch lessened this impact compared to unmodified starch.
9. Compositions containing modified starch were characterized by satisfactory resistance to thermo-oxidative aging.
10. The introduction of chemically modified starch increased the storage modulus. Samples containing a higher amount of silanized starch exhibited stronger elastic interactions and greater energy storage capacity. Composites with modified starch showed higher Payne effect values due to the formation of a stronger spatial network between the filler. Low values of the loss angle tangent, compared to the reference sample, indicated higher energy storage capacity and increased stiffness.
11. The surface properties of composites containing both modified and unmodified starch revealed low surface free energy and minimal polar components. This indicates strong intermolecular interactions and a lack of polar characteristics in the material. Surface free energy oscillated within the range of 15.48–17.85 mN/m.
12. Water contact angle increased with the starch content in both types of composites. This suggests that both modifications are effective; however, the composites made with silanized starch exhibited higher hydrophobicity and reduced water wettability compared to those made with native starch in the presence of silane. The NR15M vulcanizate obtained θ_w_ = 117.8°.
13. Silanized starch as a bio-based filler supports sustainable rubber composites, helping to reduce carbon footprints and meet eco-friendly manufacturing goals.
14. The enhanced mechanical, dynamic, and hydrophobic properties of silanized starch-filled composites make them ideal for a variety of applications, including seals, membranes, footwear, eco-friendly car tires, conveyor belts, and vibrational dampers. Moreover, the increased hydrophobicity and reduced water wettability of silanized starch-filled composites render them suitable for applications resistance to moisture.

## Figures and Tables

**Table 1:** Formulations of NR compounds.

Ingredients	Weight Parts (phr)						
NR	100	100	100	100	100	100	100
Stearic acid	2	2	2	2	2	2	2
ZnO	5	5	5	5	5	5	5
S_8_	2	2	2	2	2	2	2
MBT	1.5	1.5	1.5	1.5	1.5	1.5	1.5
Corn starch	-	5	10	15	-	-	-
OTMS	-	0.5	1	1.5	-	-	-
CS/OTMS	-	-	-	-	5	10	15
Determination of sample	NR	NR5S	NR10S	NR15S	NR5M	NR10M	NR15M

NR—natural rubber, ZnO—zinc oxide, S_8_—sulfur, MBT—2-mercaptobenzothiazole, OTMS—n-octadecylotrimethoxysilane, CS/OTMS—starch grafted with n-octadecylotrimethoxysilane.

**Table 2:** Comparison of FTIR spectra signals for modified and native corn starch ^a^.

Wavenumber (cm^−1^)	Infrared Band Assignment	NCS	CS/OTMS
3000–3600	O-H stretching [71]	+	+
2800–3000	CH_2_ asymmetric and symmetric stretching vibrations [46,59,72]	+	+
1640	Water adsorbed in the amorphous region [73,74]	+	−
1466	CH_2_ bending [71,75]	−	+
1189, 954	Si-O-C stretching [39,76,77]	−	+
1168	Si-O-Si stretching [78]	−	+
1149	C-O stretching [79]	+	−
1076	C-O-H bending [71]	+	+
993, 954, 571, 570	O-C stretching in anhydroglucose ring [80]	+	+
920–930	α-1,4 glycosidic linkages (C-O-C) [72,79]	+	+

^a^ Number in parentheses [] are the respective references to the band assignments; NCS—native corn starch; CS/OTMS—corn starch grafted with n-octadecylotrimethoxysilane; +—signal; −—no signal.

**Table 3:** *t*-test analysis for modified and unmodified starch (α = 0.05, *n* = 10).

Parameters	θ_D_	θ_G_	θ_W_
*t* value	25.6	22.6	39.7
*p*	<0.001	<0.001	<0.001
Significant difference	YES	YES	YES

Explanations of symbols below Figure 6.

**Table 4:** Cross-linking parameters determined by vulcanization kinetics and equilibrium swelling of NR vulcanizates filled with corn starch (cross-linked at T = 160 °C for t = 5 min).

Parameter	NR0	NR5S	NR5M	NR10S	NR10M	NR15S	NR15M
t_02_ (min)	2.36	1.99	1.93	1.89	1.87	1.9	1.72
t_90_ (min)	3.42	3.01	2.6	2.92	2.96	3	2.96
T_min_ (dNm)	0.23	0.21	0.2	0.16	0.27	0.1	0.27
T_max_ (dNm)	3.03	3.13	3.14	3.1	3.31	3.15	3.58
ΔT (dNm)	2.8	2.92	2.94	2.94	3.04	3.05	3.31
CRI (min^−1^)	94.3	98.0	149.2	97.1	91.7	90.9	80.6
Q_v_^T^ (mL/mL)	5.17 ± 0.29 ^a^	5.07 ± 0.42 ^a^	4.90 ± 0.22 ^a,b^	4.87 ± 0.09 ^a,b^	4.67 ± 0.19 ^a,b^	4.76 ± 0.03 ^a,b^	4.28 ± 0.06 ^b^
−Q_w_^T^ (mg/mg)	0.094 ± 0.006 ^a^	0.146 ± 0.002 ^b^	0.141 ± 0.003 ^b^	0.186 ± 0.003 ^c^	0.174 ± 0.001 ^d^	0.216 ± 0.004 ^e^	0.193 ± 0.007 ^c^
V_R_^T^ (-)	0.162 ± 0.008 ^a^	0.165 ± 0.012 ^a^	0.170 ± 0.006 ^a^	0.170 ± 0.003 ^a^	0.176 ± 0.006 ^a,c^	0.174 ± 0.001 ^a,c^	0.190 ± 0.002 ^c^
Q_v_^H^ (mL/mL)	2.61 ± 0.01 ^a^	2.56 ± 0.12 ^a^	2.50 ± 0.03 ^a,b^	2.56 ± 0.13 ^a^	2.30 ± 0.04 ^b,c^	2.53 ± 0.13 ^a,b^	2.11 ± 0.04 ^c^
−Q_w_^H^ (mg/mg)	0.101 ± 0.003 ^a^	0.132 ± 0.010 ^b^	0.119 ± 0.004 ^b^	0.179 ± 0.008 ^c^	0.169 ± 0.001 ^c^	0.214 ± 0.004 ^d^	0.205 ± 0.004 ^d^
V_R_^H^ (-)	0.277 ± 0.001 ^a^	0.281 ± 0.009 ^a^	0.286 ± 0.002 ^a,b^	0.281 ± 0.010 ^a^	0.303 ± 0.004 ^b,c^	0.284 ± 0.011 ^a,b^	0.322 ± 0.005 ^c^
Q_v_^M^ (mL/mL)	0.077 ± 0.008 ^a^	0.087 ± 0.003 ^a,b^	0.083 ± 0.007 ^a,b^	0.120 ± 0.009 ^c^	0.094 ± 0.008 ^b^	0.152 ± 0.007 ^d^	0.114 ± 0.003 ^c^
−Q_w_^M^ (mg/mg)	0.068 ± 0.001 ^a^	0.118 ± 0.003 ^b^	0.119 ± 0.010 ^b^	0.156 ± 0.005 ^c,e^	0.144 ± 0.007 ^c^	0.185 ± 0.006 ^d^	0.172 ± 0.006 ^d,e^
V_R_^M^ (-)	0.929 ± 0.006 ^a^	0.920 ± 0.002 ^a,b^	0.923 ± 0.005 ^a,b^	0.893 ± 0.007 ^c^	0.914 ± 0.007 ^b^	0.868 ± 0.005 ^d^	0.898 ± 0.002 ^c^
α_c_ (-)	0.194 ± 0.011 ^a^	0.198 ± 0.017 ^a^	0.204 ± 0.009 ^a^	0.205 ± 0.004 ^a^	0.214 ± 0.009 ^a,b^	0.210 ± 0.002 ^a,b^	0.235 ± 0.003 ^b^

t_02_—scorch time; t_90_—vulcanization time; T_min_—minimum torque; T_max_—maximum torque; ΔT—torque increment; CRI—cure rate index; Q_v_—equilibrium volumetric swelling: in toluene (Q_v_^T^), hexane (Q_v_^H^), or methanol (Q_v_^M^); −Q_w_—the content of the eluted fraction: in toluene (−Q_w_^T^), hexane (−Q_w_^H^), or methanol (−Q_w_^M^); V_R_—volume fraction of rubber in swollen material: in toluene (V_R_^T^), hexane (V_R_^H^), or methanol (V_R_^M^); α_c_—degree of cross-linking. Superscript letters indicate statistically homogeneous subsets (Tukey’s HSD test; α = 0.05).

**Table 5:** Comparison of tensile strength before and after thermo-oxidative aging (T = 70 °C; t = 7 days) of NR vulcanizates containing corn starch (cross-linked at T = 160 °C for t = 5 min).

Parameter	S_E100_ (MPa)	S′_E100_ (MPa)	TS_b_ (MPa)	TS′_b_ (MPa)	E_b_ (%)	E′_b_ (%)	AF (-)
NR0	0.560 ± 0.014 ^a^	0.690 ± 0.032 ^a,b,c^	10.6 ± 0.4 ^a,c^	16.2 ± 0.5 ^a^	1284 ± 3	1273 ± 13	1.45
NR5S	0.583 ± 0.010 ^a^	0.682 ± 0.037 ^b^	10.6 ± 0.6 ^a,b,c^	13.1 ± 1.1 ^b^	1285 ± 2	1207 ± 54	1.18
NR5M	0.627 ± 0.031 ^b^	0.681 ± 0.015 ^a,b,c^	11.0 ± 1.0 ^c^	16.1 ± 1.5 ^a^	1265 ± 34	1270 ± 19	1.62
NR10S	0.574 ± 0.024 ^a^	0.654 ± 0.035 ^a,b^	9.8 ± 0.6 ^a,d^	11.9 ± 0.7 ^b^	1284 ± 2	1284 ± 2	1.17
NR10M	0.633 ± 0.016 ^b,c^	0.685 ± 0.010 ^a,b,c^	11.4 ± 0.6 ^b,c^	14.3 ± 0.7 ^c^	1285 ± 4	1285 ± 3	1.25
NR15S	0.605 ± 0.039 ^a,b^	0.717 ± 0.020 ^c^	9.1 ± 0.8 ^d^	12.4 ± 1.2 ^b^	1224 ± 68	1195 ± 47	1.33
NR15M	0.668 ± 0.002 ^c^	0.765 ± 0.005 ^d^	11.4 ± 0.3 ^b,c^	15.8 ± 0.5 ^a,c^	1280 ± 10	1275 ± 21	1.38

S_E100_—stress at an elongation of 100%; TS_b_—tensile strength; E_b_—elongation at break; S′_E100_—stress at an elongation of 100% after thermo-oxidative aging; TS′_b_—tensile strength after thermo-oxidative aging; E′_b_—elongation at break after thermo-oxidative aging; AF—aging factor. Superscript letters indicate statistically homogeneous subsets (Tukey’s HSD test, α = 0.05).

**Table 6:** Other mechanical properties of tested NR composites (cross-linked at T = 160 °C for t = 5 min).

Parameter	T_R_ (N/mm)	HA (°ShA)	ΔW_1_ (N⸳mm)	ΔW_5_ (N⸳mm)	E_M_ (%)
NR0	6.96 ± 0.45 ^a^	45.8 ± 1.8 ^a^	21.67 ± 1.41 ^a^	17.09 ± 0.65 ^a^	8 ± 1 ^a^
NR5S	6.24 ± 0.44 ^a,b^	47.8 ± 2.0 ^a^	24.82 ± 0.48 ^a,b^	18.04 ± 0.28 ^a,b^	10 ± 1 ^a^
NR5M	5.58 ± 0.25 ^b,c^	52.1 ± 2.3 ^b^	25.20 ± 0.40 ^a,b^	18.91 ± 0.14 ^a,b^	9 ± 1 ^a^
NR10S	5.10 ± 0.27 ^c,d^	52.5 ± 1.6 ^b,c^	27.75 ± 2.11 ^b,c^	18.58 ± 1.40 ^a,b^	13 ± 1 ^b^
NR10M	6.19 ± 0.21 ^b^	55.4 ± 2.3 ^c,d^	29.35 ± 0.65 ^c,d^	19.68 ± 0.39 ^b^	13 ± 1 ^b,c^
NR15S	4.47 ± 0.06 ^d^	56.2 ± 2.4 ^d^	31.24 ± 0.82 ^d^	18.89 ± 0.84 ^a,b^	17 ± 1 ^d^
NR15M	5.23 ± 0.45 ^c^	60.2 ± 2.9 ^e^	29.52 ± 1.34 ^c,d^	18.44 ± 0.31 ^a,b^	16 ± 2 ^c,d^

T_R_—tear resistance; HA—shore A hardness; ΔW_1_, ΔW_5_—hysteresis losses during the first and fifth sample stretching cycles; E_M_—Mullins effect. Superscript letters indicate statistically homogeneous subsets (Tukey’s HSD test, α = 0.05).

**Table 7:** Dynamic properties of tested NR composites (cross-linked at T = 160 °C for t = 5 min).

Parameter	G′_max_ (kPa)	G″_max_ (kPa)	ΔG’ (kPa)	tan(δ) (-)
NR0	131.9	29.5	97.0	1.07
NR5S	139.4	21.6	105.2	0.48
NR5M	160.7	23.1	131.3	0.39
NR10S	183.8	32.5	152.1	0.43
NR10M	207.6	17.6	158.9	0.22
NR15S	213.7	29.1	177.1	0.43
NR15M	241.1	41.2	203.8	0.75

G′_max_—maximum storage modulus; G″_max_—maximum loss modulus; ΔG′—Payne effect; tan(δ)—maximum loss tangent.

**Table 8:** Comparison of the type of starch modification for selected properties of NR composites (cross-linked at T = 160 °C for t = 5 min).

Properties		Content of Starch		
	Statistical Parameters	5	10	15
S_E100_	*t* value	3.86	5.31	4.68
	*p*	0.005	<0.001	0.002
	Significant difference	YES	YES	YES
S′_E100_	*t* value	0.77	2.43	7.17
	*p*	0.461	0.041	<0.001
	Significant difference	NO	YES	YES
TS_b_	*t* value	0.86	4.94	6.52
	*p*	0.417	0.001	<0.001
	Significant difference	NO	YES	YES
TS′_b_	*t* value	4.43	5.74	7.22
	*p*	0.003	<0.001	<0.001
	Significant difference	YES	YES	YES
T_R_	*t* value	2.6	6.24	3.93
	*p*	0.041	<0.001	0.006
	Significant difference	YES	YES	YES
HA	*t* value	4.34	3.18	3.26
	*p*	<0.001	0.005	0.004
	Significant difference	YES	YES	YES
E_M_	*t* value	3.41	0.32	1.22
	*p*	0.042	0.762	0.289
	Significant difference	YES	NO	NO
α_c_	*t* value	0.54	1.58	12.00
	*p*	0.618	0.189	<0.001
	Significant difference	NO	NO	YES
θ_w_	*t* value	4.11	4.22	4.49
	*p*	<0.001	<0.001	<0.001
	Significant difference	YES	YES	YES

## Data Availability

The original contributions presented in the study are included in the article, and further inquiries can be directed to the corresponding author.
